# Real-world study of multiple naloxone administration for opioid overdose reversal among bystanders

**DOI:** 10.1186/s12954-022-00627-3

**Published:** 2022-05-20

**Authors:** Randa Abdelal, A. Raja Banerjee, Suzanne Carlberg-Racich, Neyla Darwaza, Diane Ito, Jessica Shoaff, Josh Epstein

**Affiliations:** 1 Hikma Pharmaceuticals USA Inc., 200 Connell Drive, 4th Floor, Berkeley Heights, NJ 07922 USA; 2 Hikma Specialty USA Inc., Berkeley Heights, NJ USA; 3 DePaul University, Chicago, IL USA; 4 Hikma Pharmaceuticals LLC, Amman, Jordan; 5 Stratevi, LLC, Santa Monica, CA USA; 6 Stratevi, LLC, Boston, MA USA

**Keywords:** Naloxone, Narcan, Multiple naloxone administrations, Dosing, Opioid overdose, Fentanyl, Synthetic opioid, Bystander, Layperson, COVID-19

## Abstract

**Background:**

The increasing prevalence of highly potent, illicitly manufactured fentanyl and its analogues (IMF) in the USA is exacerbating the opioid epidemic which has worsened during the COVID-19 pandemic. Narcan® (naloxone HCl) Nasal Spray has been approved by the US Food and Drug Administration as a treatment for opioid-related overdoses. Due to the high potency of IMF, multiple naloxone administrations (MNA) may be needed per overdose event. It is essential to determine the patterns of naloxone use, including MNA, and preferences among bystanders who have used naloxone for opioid overdose reversal.

**Methods:**

A cross-sectional web-based survey was administered to 125 adult US residents who administered 4 mg Narcan® Nasal Spray during an opioid overdose in the past year. The survey asked about the most recent overdose event, the use of Narcan® during the event and the associated withdrawal symptoms, and participant preferences regarding dosages of naloxone nasal spray. An open-ended voice survey was completed by 35 participants.

**Results:**

Participants were mostly female (70%) and white (78%), while reported overdose events most frequently occurred in people who were males (54%) and white (86%). Most events (95%) were successfully reversed, with 78% using ≥ 2 doses and 30% using ≥ 3 doses of Narcan® Nasal Spray. Over 90% were worried that 1 Narcan® box may not be enough for a successful future reversal. Reported withdrawal symptoms were similar in overdose events where 1 versus ≥ 2 sprays were given. Eighty-six percent of participants reported more confidence in an 8 mg versus a 4 mg naloxone nasal spray and 77% reported a stronger preference for 8 mg over 4 mg.

**Conclusions:**

MNA occurred in most overdose events, often involving more sprays than are provided in one Narcan® nasal spray box, and participants predominantly expressed having a stronger preference for and confidence in an 8 mg compared to a 4 mg nasal spray. This suggests the need and desire for a higher dose naloxone nasal spray formulation option. Given that bystanders may be the first to administer naloxone to someone experiencing an opioid overdose, ensuring access to an adequate naloxone supply is critical in addressing the opioid overdose epidemic.

## Background

The opioid overdose epidemic is a significant public health issue in the USA where the rate of opioid overdose deaths continues to rise exponentially. In 2019, there were approximately 50,000 deaths due to opioid overdoses [1], representing 70–80% of all drug overdose deaths in the USA [1, 2]. The opioid overdose epidemic has been further exacerbated by the COVID-19 pandemic; recent data suggests an increase in nonprescription opioid use during the pandemic with the percent of tested individuals screening positive for nonprescription fentanyl and heroin increasing by 35% and 44%, respectively [3]. Additionally, preliminary data released by the CDC revealed a 37% increase in opioid overdose deaths in 2020 compared to 2019; opioids were involved in nearly 70,000 deaths representing 75% of the all overdose deaths [4].

Opioids are a large class of drugs encompassing both prescription medications (including morphine, codeine and fentanyl) and illegal drugs (including heroin and illicitly manufactured synthetic opioids). According to the Centers for Disease Control and Prevention (CDC), the majority of opioid-related overdose deaths involve the use of illicitly manufactured fentanyl (IMF), a synthetic opioid considered to be up to 100 times more potent than morphine [2]. While the IMF epidemic is well documented in the USA, evidence is emerging of significant growth of IMF-related deaths in European countries [5, 6].

Naloxone has been shown to be effective in rapidly reversing opioid overdose and specifically, Narcan® [naloxone hydrochloride] has been approved by the US Food and Drug Administration (FDA) as a safe and effective treatment for opioid-related overdoses [7]. A box of Narcan® contains 2 4 mg doses of naloxone. In accordance with the Narcan® label, multiple doses of naloxone may be administered if the individual does not respond to the first dose or relapses into respiratory depression [7]. Due to the increased prevalence of fentanyl overdoses, the CDC has issued health alerts highlighting that multiple naloxone administrations (MNA) may be needed per overdose event due to the high potency of IMF [8]. Indeed, data suggest that the need for MNA for successful revivals of opioid overdoses is increasing [9]; findings from a recent targeted literature review of MNA rates in the USA suggest that administering two or more naloxone doses is not uncommon in both community and emergency medical services settings and the frequency is increasing [10–14].

In opioid overdose emergencies, bystanders are often the first to witness or be in the presence of the person experiencing an overdose. Approximately 40% of overdose deaths reported to the CDC State Unintentional Drug Overdose Reporting System (SUDORS) between January–June 2019 occurred in the presence of a bystander [15]. Since 1996, community-based harm reduction programs have provided an increasing number of naloxone take-home kits to laypersons [16], a strategy that has been found to successfully reduce opioid overdose deaths [17–19]. While individual kits may vary, a typical naloxone take-home kit contains 2–3 doses of either 0.4 mg intramuscular (IM) or 4 mg of intranasal (IN) naloxone. Several recent studies have reported that MNA occurs frequently in community settings, with reported rates of bystander MNA ranging from 43 to 83% [20–23].

Determining the patterns of naloxone use, including MNA, from the perspective of bystanders is essential given the rise in naloxone administration from bystanders and the challenges in capturing accurate data on this stigmatized and often isolated population. Furthermore, gaining insight into the overdose reversal experiences and preferences among those who have administered naloxone for opioid overdose reversal is critical to better assess the unmet needs among those at risk of an opioid overdose. As such, the objectives of this study were to gather real-world evidence to characterize the circumstances of the overdose event and the patterns of intranasal naloxone use among bystanders who have administered naloxone during an opioid overdose in the past year, to describe the naloxone-associated acute opioid withdrawal symptoms experienced after the revival attempt and to identify participants’ naloxone dosing preferences.

## Methods

A web-based, cross-sectional survey was administered to 125 adults who reported witnessing an overdose event and administering 4 mg Narcan® Nasal Spray to the person who overdosed in the past 12 months. Additionally, a subset of 35 participants were asked via automated voice response about their experiences administering 4 mg Narcan® Nasal Spray during an overdose event and preferences between nasal spray dosages. Participants were eligible to participate if they were at least 18 years old, had administered 4 mg Narcan® Nasal Spray to someone experiencing an opioid overdose event in the past 12 months, were able to read and understand English and had access to a cell phone or computer with internet access. Participants were also required to reside in a region of the USA with higher IMF use (Northeast, Midwest or South, based on US Census Regions), defined as > 50% of opioid mortalities involving a synthetic opioid based on data from the 2018 CDC Wide-ranging Online Data for Epidemiologic Research (WONDER) database. Participants were primarily recruited through existing patient panels and were invited to complete an eligibility screener to determine if they qualified for the study. Individuals who met study eligibility criteria were invited to participate. Centralized institutional review board (IRB) approval for this study was obtained by New England Independent Review Board with all participants providing electronic informed consent to participate in the study. The study was opened on February 15, 2021, and closed on March 26, 2021. Additionally, participants received an honorarium in the form of a $50 Walmart gift certificate upon completion of the online survey and an additional $25 Walmart gift certificate for completing the open-ended voice response questions.

The study was executed utilizing a proprietary technology platform that enables the collection of data via web-enabled surveys and automated voice response. Both the web-enabled survey questions and open-ended voice response questions were developed by the research team with a 7th grade reading level to ensure readability and comprehension among study participants [24]. To maximize data quality, participant responses were reviewed for accuracy by screening for unrealistic survey completion times and for patterned, nonsensical or inconsistent responses. To further minimize data entry error, the following validation checks were programmed into the survey platform: skip patterns so participants only received relevant questions, out of range values and custom logic to verify that the number of sprays given by the participant and others added up to the total number sprays administered.

The web-enabled survey captured participant demographics, characteristics of the overdose event and the person who overdosed and details about the use of 4 mg Narcan® Nasal Spray during the overdose event. The specific questions pertaining to the overdose event and use of Narcan® included the following: location of overdose, relationship to the person who overdosed, number of 4 mg Narcan® Nasal Sprays administered during the overdose event, approximate time waited between sprays given, number of doses of 4 mg Narcan® Nasal Spray left over, drugs the person who overdosed thought they were taking, perception of whether or not fentanyl or fentanyl-like drug was involved in the overdose, was 911 call made and outcomes for the person who overdosed, including possible opioid withdrawal symptoms. The survey also captured information on participant perceptions about IMF in the local drug supply and asked respondents to compare a 4 mg and an 8 mg naloxone nasal spray on the following domains: concerns about opioid withdrawal, level of confidence to revive someone and the preferred dose to revive someone from an opioid overdose. Participants reporting more than one event in the past 12 months were asked to respond to the most recent overdose in which they administered Narcan® Nasal Spray.

The open-ended voice response questions covered the following topics: the perceptions and feelings of witnessing an overdose event, details regarding the number of Narcan® Nasal Spray doses administered during the overdose event, perceptions of the effectiveness of Narcan® Nasal Spray in treating the overdose, degree of participant confidence that Narcan will help in a future overdose situation, participant preferences on having an 8 mg nasal spray available and potential use of an 8 mg nasal spray if it made opioid withdrawal worse.

Descriptive statistics were used to summarize the survey responses. Additionally, linguistic and thematic analyses were conducted on the voice response data.

## Results

### Participant characteristics

The demographic characteristics of the 125 study participants are presented in Table 1. Participants were predominantly female (*n* = 88, 70%) and white (*n* = 97, 78%) with a mean age of 41 (Standard Deviation ± 9.7) years. Most study participants (*n* = 76, 61%) reported administering Narcan® Nasal Spray at more than one overdose event in the past 12 months and 35% (*n* = 44) have had an opioid overdose themselves.

**Table 1:** Characteristics of 125 study participants and overdose events

Participant characteristics (*N* = 125)	
Age (years)	
Mean (SD)	40.9 (9.7)
Median (range)	40 (18–68)
Gender [*n* (%)]	
Female	88 (70%)
Male	37 (30%)
Race/ethnicity [*n* (%)]	
White	97 (78%)
Mixed race	13 (10%)
Black	6 (5%)
Asian	5 (4%)
Hispanic	3 (2%)
Other	1 (1%)
Type of residential community [*n* (%)]	
Suburban	54 (43%)
Rural	38 (30%)
Urban	33 (27%)
Number of overdose events in the past 12 months where participant has administered Narcan® Nasal Spray [*n* (%)]	
1	49 (39%)
2	41 (33%)
3	14 (11%)
≥ 4	21 (17%)
Experienced an overdose themselves [*n* (%)]	
Yes	44 (35%)
No	81 (65%)

### Characteristics of the person who overdosed and the overdose event

Of the 125 reported overdose events, the people who overdosed were predominantly male (*n* = 68, 54%) and white (*n* = 108, 86%) with a mean age of 34 (± 11) years, as reported by study participants (Table 2). Compared to the Mortality Disparities in American Communities Study (MDAC), a large nationally representative database of individuals who experienced a fatal opioid overdose, this study sample was similar in terms of gender, race/ethnicity and age [25]. Emergency services (i.e., 911) were reported as called for 71% (*n* = 89) of the overdose events and 95% (*n* = 119) of the revival attempts were successful. The reported overdose events frequently happened to someone that the participant knew (*n* = 110, 88%) and often occurred in a home belonging to the participant (*n* = 31, 25%) or someone else (*n* = 56, 45%) (Table 2).

**Table 2:** Characteristics of the people who overdosed and the overdose events (*N* = 125)

Characteristics of the person who overdosed (*N* = 125)	
Age (years)	
Mean (SD)	34.2 (10.5)
Median (Range)	32 (17–76)
Gender [*n* (%)]	
Female	56 (45%)
Male	68 (54%)
Unknown	1 (1%)
Race/ethnicity [*n* (%)]	
White	108 (86%)
Black	9 (7%)
Mixed race	4 (3%)
Hispanic	2 (2%)
Asian	0 (0%)
Other	2 (2%)

Characteristics of the overdose event (*N* = 125)	
Outcome of revival attempt	
Revived at the scene	110 (88%)
Revived later	9 (7%)
Not revived	6 (5%)
Emergency services (911) called	
Yes	89 (71%)
No	35 (28%)
Unknown	1 (1%)
Overdose location	
Someone else’s home	56 (45%)
Participant’s home	31 (25%)
Outside	16 (13%)
Vehicle	9 (7%)
Other* (hotel/motel, place of business, short-term housing)	13 (10%)
Relationship to study participant	
Friend	49 (39%)
Family member	31 (25%)
Acquaintance	21 (17%)
Stranger	13 (10%)
Significant other/Spouse	9 (7%)
Other	2 (2%)
Number of drugs the person who overdosed thought they were taking	
1	64 (51%)
2	44 (35%)
3+	15 (12%)
Unknown	2 (2%)

Participants reported that the Narcan® Nasal Spray they used during the overdose event came from a community group, substance use treatment center, hospital/clinic or other social service agency (*n* = 55, 44%), the person who overdosed (*n* = 28, 22%), a prescription or purchased over the counter at pharmacy (*n* = 26, 21%) or someone else (*n* = 14, 11%).

Of the 82 participants (66%) who had their own Narcan® Nasal Spray on hand for the overdose event, 71% (*n* = 58) reported having multiple doses on hand (mean = 3.0 ± 0.3). Of those participants, 55% (*n* = 45) reported having no sprays left over after the revival attempt (mean: 0.7 (0.1)).

**Fig. 1:**
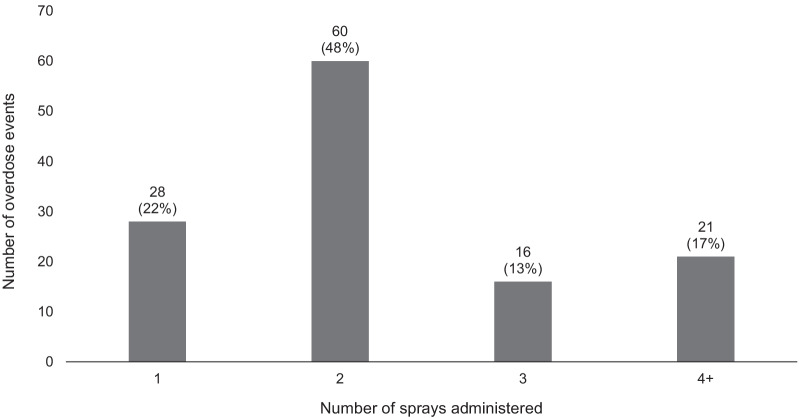
Number of Narcan® Nasal Sprays administered during overdose event (*N* = 125)

Multiple doses of Narcan® Nasal Spray were administered during most of the overdose events (mean: 2.4 ± 0.1); two or more doses of Narcan® Nasal Spray were administered in 78% (*n* = 97) of overdose events, while three or more were administered in 30% (*n* = 37) of events (Fig. 1). Participants themselves most frequently administered one spray (58%, *n* = 72) and additional sprays were administered by other people in 62% (*n* = 60) of overdose events where more than one spray was given. Of the 53 participants who administered multiple doses of Narcan® Nasal Spray themselves, 68% (*n* = 36) reported waiting between administering sprays while 30% (*n* = 16) reported that they immediately gave the additional spray(s).

According to study participants, the people who overdosed most frequently believed they were taking heroin (66%, *n* = 83) (Table 3). Additionally, 47% (*n* = 59) of the people who overdosed thought they were taking more than one drug (Table 2). Among those taking more than one drug, the most common combinations were mixtures of opioids including heroin, nonprescription fentanyl or opioid pills (26% of overdose events, *n* = 32) or mixtures of opioids and benzodiazepine/tranquilizers (18% of overdose events, *n* = 22). According to participants, fentanyl was believed to be involved in 73% (*n* = 91) of the overdose events.

**Table 3:** Participant reports of what drugs the person who overdosed thought they were taking (*N* = 125)

Drug	*n* (%)
Heroin	83 (66%)
Nonprescription fentanyl or fentanyl-like drug	37 (30%)
Opioid pills prescribed to the person who overdosed (such as OxyContin, Vicodin and Actiq)	24 (19%)
Benzodiazepines/tranquilizers (such as Xanax, Valium or Ativan)	22 (18%)
Opioid pills acquired elsewhere (such as OxyContin, Vicodin and Actiq)	17 (14%)
Cocaine or other stimulants	13 (10%)
Alcohol	9 (7%)
Marijuana	5 (4%)
Other	2 (2%)

### Withdrawal-associated symptoms

Withdrawal-associated symptoms experienced by the people who overdosed are presented in Table 3. As reported by study participants, 98% (*n* = 123) of the people who overdosed experienced at least one withdrawal-associated symptom, with the most common symptoms reported as confusion (*n* = 76, 61%), body weakness (*n* = 63, 50%) and agitation/anger (*n* = 63, 50%). Withdrawal-associated symptoms were mostly similar in people receiving 1 versus ≥ 2 doses of Narcan® Nasal Spray (Table 4).

**Table 4:** Participant reported withdrawal symptoms experienced by the people who overdosed (*N* = 125)

Symptom	Percent of participants experiencing the symptom [*n* (%)]		
	Overall (*N* = 125)	1 spray administered (*n* = 28)	≥ 2 sprays administered (*n* = 97)
Confusion	76 (61%)	17 (61%)	59 (61%)
Agitated/Angry	63 (50%)	15 (54%)	48 (49%)
Body weakness	63 (50%)	13 (46%)	50 (52%)
Nausea	61 (49%)	13 (46%)	48 (49%)
Shivering	58 (46%)	14 (50%)	44 (45%)
Sweating	52 (42%)	12 (43%)	40 (41%)
Headaches	50 (40%)	8 (29%)	42 (43%)
Dizziness	46 (37%)	8 (29%)	38 (39%)
Vomiting	42 (34%)	10 (36%)	32 (33%)
Body pain	26 (21%)	6 (21%)	20 (21%)
Nasal congestion	15 (12%)	4 (14%)	11 (11%)
Nasal dryness	15 (12%)	0 (0%)	15 (15%)
Yawning	13 (10%)	2 (7%)	11 (11%)
Other	11 (9%)	1 (4%)	10 (10%)
None	2 (2%)	0 (0%)	2 (2%)

### Participant naloxone dosing concerns and preferences

**Fig. 2:**
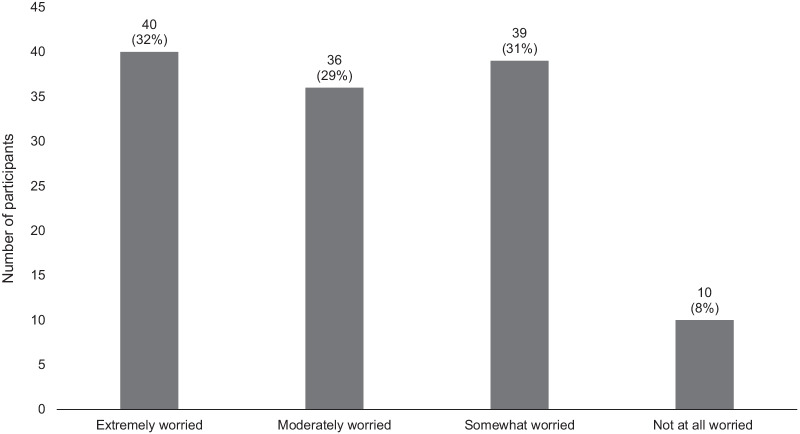
Participant worry that one box of Narcan® Nasal Spray may not be enough to successfully reverse an opioid overdose (*N* = 125)

**Fig. 3:**
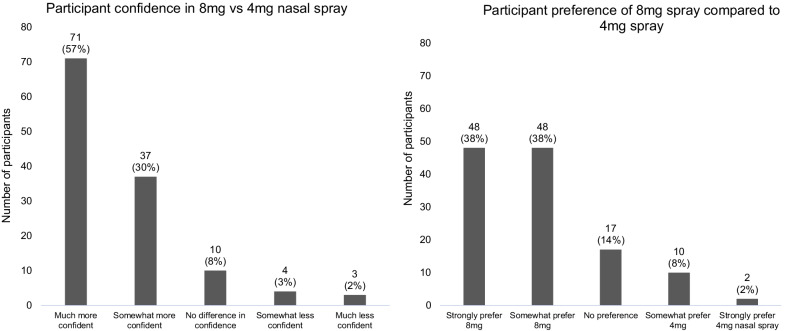
Participant preference and confidence in an 8 mg compared to a 4 mg nasal spray (*N* = 125)

**Fig. 4:**
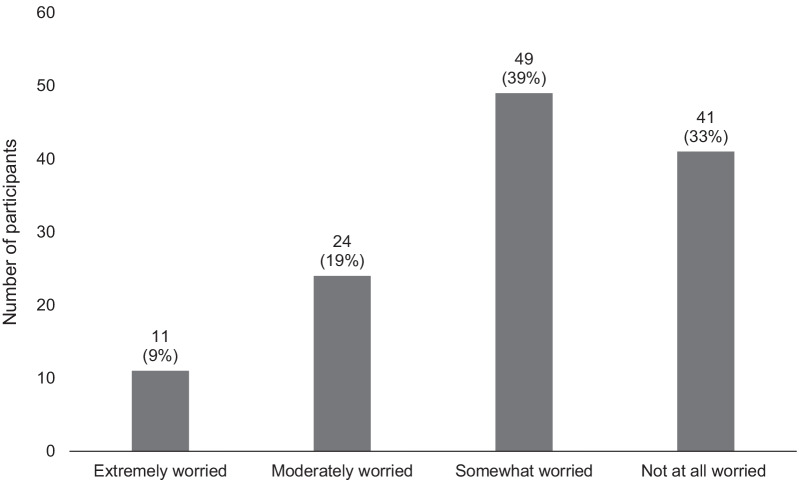
Participant worry about withdrawal symptoms from an 8 mg compared to a 4 mg nasal spray (*N* = 125)

Most study participants believe that the drug supply in their community is mixed with some form of IMF to a moderate (*n* = 48, 38%) or great (*n* = 50, 40%) extent. The vast majority of participants (92%) reported being worried that having one box of Narcan® Nasal Spray on hand may not be enough to revive someone from an opioid overdose, with approximately one third of participants each reporting being extremely (*n* = 40, 32%), moderately (*n* = 36, 29%) or somewhat (*n* = 39, 31%) worried, while only 8% (*n* = 10) were not worried at all (Fig. 2). If given the choice, 86% (*n* = 108) of participants would feel more confident reviving someone with an 8 mg (mg) nasal spray compared to a 4 mg nasal spray (Fig. 3). Similarly, 77% (*n* = 96) of participants would prefer to have an 8 mg compared to a 4 mg nasal spray on hand to revive someone from an opioid overdose (Fig. 3). Only 28% (*n* = 35) of participants reported being extremely (*n* = 11, 9%) or moderately (*n* = 24, 19%) worried about someone going through withdrawal because they received an 8 mg nasal spray compared to a 4 mg nasal spray, while 39% (*n* = 49) and 33% (*n* = 41) reported being somewhat worried or not worried at all, respectively (Fig. 4).

### Voice responses

**Fig. 5:**
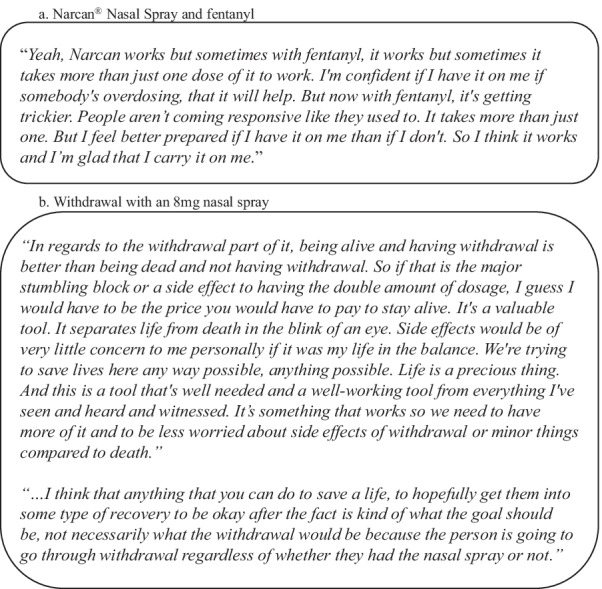
Participant voice responses (*N* = 3)

The subset of 35 participants who completed the open-ended voice response survey routinely described feeling panicked during the overdose event, followed by relief and gratitude after a successful revival attempt. While most expressed belief that Narcan® Nasal Spray would work in future opioid overdose reversals, several expressed concerns that a 4 mg dose may be insufficient to successfully revive someone. Several participants also noted a change in how individuals respond to Narcan® Nasal Spray when it is suspected they have some form of fentanyl in their system, stating that they need to use more sprays to revive these individuals (Fig. 5).

A majority of the 35 participants who completed the voice response survey expressed a strong preference for an 8 mg naloxone nasal spray compared to a 4 mg nasal spray, with most stating the belief that an 8 mg option would be more effective and save more lives. There were only 4 participants who indicated that they would not use an 8 mg option. While some participants expressed concern that an 8 mg nasal spray may result in more severe withdrawal symptoms than a 4 mg option, most conveyed that this would not diminish their preference in a stronger dose as they prioritize saving a life over avoiding withdrawal symptoms (Fig. 5). Several participants further elaborated that withdrawal after a reversal attempt is always a possibility, and they do not consider it a barrier to using an 8 mg dose (Fig. 5).

## Discussion

This real-world study of bystander administration of naloxone for opioid overdose events found that MNA occurred in most overdose events; two or more doses were administered in 78% of overdose events, while 3 or more doses were administered in 30% of events. These findings are similar to results from a recent survey of people who use opioids, which found that 79% of overdose events responded to by community members required two or more doses of naloxone to reverse the overdose [23]. These recent estimates are a notable increase from what was reported in a briefing report of a FDA Advisory Committee meeting in September 2016 where data collected from eight community organizations found that two or more doses Narcan® Nasal Spray were used in only 32% of community-based revival attempts [26]. This recent trend of dramatically increasing MNA rates in community settings corresponds with the significant increase of IMF in the drug supply as well as the rise of opioid-related overdoses which have escalated during the COVID-19 pandemic. As community distribution of naloxone is an important strategy to reduce opioid overdose deaths, determining how much naloxone is being administered by bystanders who are often the first to render aid to someone experiencing an opioid overdose is critical.

Notably, this study also found that slightly more than half of study participants who had Narcan® Nasal Spray on hand for the overdose event did not have any left over after the revival attempt and that additional doses from another person were administered in 48% (*n* = 60) of the overdose events. Together, these results highlight the need for multiple doses of Narcan® Nasal Spray in overdose events and underscore the importance of naloxone distribution in high-risk communities. These findings also suggest that the typical naloxone kits (which often contain two 4 mg doses per kit) that are frequently distributed to community members [15, 17] may not be sufficient for many overdose events. Among participants of this study, community organizations were the primary source of Narcan® Nasal Spray, highlighting the importance of the distribution efforts by these groups. For organizations unable to provide multiple Narcan® Nasal Spray kits per person, a nasal spray with a higher dose (i.e., 8 mg instead of 4 mg) may be a reasonable solution.

Further, most study participants reported a stronger preference for an 8 mg, compared to a 4 mg nasal spray, and similarly reported having more confidence in successfully reviving someone from an opioid overdose with an 8 mg compared to a 4 mg nasal spray. Additionally, that vast majority of participants expressed concern that one box of Narcan® Nasal Spray may not be enough to revive someone from an opioid overdose. The perceived need for a stronger naloxone formulation may be related to the prevalence of IMF in participants’ communities, as 78% of participants believe that IMF is present in their community drug supply to a moderate or great extent. In voice responses, participants frequently expressed the belief that an 8 mg spray would be more effective at saving lives than a 4 mg spray, and posited that an 8 mg spray may work faster at reversing an overdose than a 4 mg spray and that fewer sprays would need to be administered. Many also noted that an 8 mg spray might allow them to carry fewer sprays.

While withdrawal symptoms were reported in all but two of the overdose events, the frequency of symptoms were similar among people who received one versus two or more doses of Narcan® Nasal Spray. This is consistent with what was reported in the previously described briefing report of a FDA Advisory Committee meeting in September 2016 which found equal proportions of withdrawal symptoms between cases who were administered one versus two doses of Narcan Nasal Spray [26]. As noted in the FDA Advisory Committee meeting, naloxone-associated withdrawal side effects are rarely life threatening and the risks of withdrawal symptoms should be weighed against the risk of death from the opioid overdose [26]. This was a common sentiment throughout participant voice responses as well, where most participants conveyed that surviving an overdose event is more important than avoiding transient withdrawal symptoms.

These results should be interpreted in the context of several limitations. First, the sample size of the study was small and participants were fairly homogeneous. Results may not be generalizable, particularly to the western part of the country which was outside of the study region or to nonwhite community members. Additionally, while there are often barriers to calling emergency services due to fear of being arrested for use or possession of illegal drugs, emergency services were called for a majority (71%) of the overdose events reported by study participants. This potentially indicates that our study population represents a group who feel comfortable/safe interacting with emergency personnel. Despite the high frequency of 911 calls by this study population, in open-ended voice responses some participants mentioned that they administered Narcan® Nasal Spray to avoid calling 911. It is possible that in communities that are more fearful of contacting emergency services, there will be an even stronger demand for higher dosage options.

Additionally, as this study was based on participants observations of overdose events, the reported withdrawal symptoms only reflect what was observed by the participant and may not precisely reflect what was experienced by the person who overdosed. Similarly, information about what drugs the person who overdosed thought they were taking are based on the participants’ secondhand report. We were unable to assess whether more than one dose of Narcan® Nasal Spray was truly necessary in the overdose events where more than one dose was administered. Despite this, our results demonstrate the high frequency of MNA in the community setting and the desire for stronger doses by community members.

Since the completion of this study, a new 8 mg naloxone nasal spray, Kloxxado™ (naloxone hydrochloride) has been approved by the FDA to reverse the effects of opioid overdose. Future research should be initiated to better assess the MNA rates for opioid overdoses among the various treatment options.

## Conclusions

MNA occurred in most overdose events, often involving more sprays than are provided in one Narcan® box, and study participants predominantly expressed a stronger preference for and more confidence in an 8 mg compared to a 4 mg nasal spray. This suggests the need and desire for a higher dose naloxone nasal spray formulation option. Given that bystanders may be the first to administer naloxone to someone experiencing an opioid overdose, ensuring access to an adequate supply is critical in preventing opioid-related deaths.

## Data Availability

The data analyzed in this study are not publicly available.
